# P-1478. Trends in Streptococcus pneumoniae serotypes and antimicrobial resistance among US adults ≥18 years old with invasive and noninvasive pneumococcal disease (2022-2023)

**DOI:** 10.1093/ofid/ofaf695.1664

**Published:** 2026-01-11

**Authors:** Mekki Bensaci, Karri A A Bauer, Kenneth Klinker, Jason Cota, Pavel Prusakov, Rodrigo E Mendes, Kristen Feemster

**Affiliations:** Merck & Co., Inc., Kenilworth, New Jersey; Merck & Co, Inc, Kenilworth, New Jersey; Merck & Co, Inc, Kenilworth, New Jersey; Merck, Upper Gwayned, New Jersey; Merck, Upper Gwayned, New Jersey; Element Iowa City (JMI Laboratories), North Liberty, IA; Merck, Upper Gwayned, New Jersey

## Abstract

**Background:**

Antimicrobial resistance (AMR) is a global public threat. Pneumococcal conjugate vaccines (PCVs) contribute both directly and indirectly to combating AMR. The US Advisory Committee on Immunization Practices (ACIP) recommends PCV21 for adults ≥50 years (yrs). PCV21 provides serotype (ST) coverage for 83% of invasive pneumococcal disease (IPD) with 30% of cases caused by eight STs not included in other licensed vaccines. It is important to monitor *S. pneumoniae* epidemiology and AMR trends to understand the impact of new PCVs. We evaluated ST distribution and antimicrobial susceptibility of *S. pneumoniae* obtained from adult patients (≥18 yrs) with invasive pneumococcal disease (IPD) and non-IPD during 2022-2023.

**Methods:**

: *S. pneumoniae* isolates causing IPD and non-IPD were collected from 30 sites in 19 states between 2022 and 2023. Whole genome sequencing of the *S. pneumoniae* isolates was performed using Illumina NextSeq sequencers (Illumina, San Diego, CA, USA). Capsular locus sequences were extracted and analyzed using the PneumoCaT database for serotype determination. Antimicrobial susceptibility tests were performed using the broth microdilution method.

**Results:**

Among the 675 *S. pneumoniae* isolates, ST3 was the most common (12.3%), followed by 35B (9.8%), 9N (6.8%), 22F (6.4%), 11A (6.1%), 19F (6.1%), 23A (5.6%), and 15A (5.2%), with PCV21 covering 83% of isolates compared to 50% for PCV20. PCV20 STs demonstrated susceptibility to penicillin (80.7%), ceftriaxone (99.4%), and azithromycin (63.5%). In contrast, PCV21 STs demonstrated lower susceptibility to penicillin (65.3%), ceftriaxone (98.8%), and azithromycin (53.7%). PCV21 unique STs demonstrated the lowest susceptibility to penicillin (52%), azithromycin (49.5%), and doxycycline (77.8%) compared to other vaccine types. Serotypes, 35B (3%), 23A (52.6%), and 23B (52.6%) demonstrated the lowest penicillin susceptibility.

**Conclusion:**

Newly introduced PCVs include several serotypes associated with high rates of AMR, particularly PCV21 whose unique serotypes had the lowest penicillin susceptibility. Thus, new PCVs have the potential to help reduce AMR. However, continued surveillance is needed to evaluate trends in the impact of PCVs on ST epidemiology and AMR over time.

**Disclosures:**

Mekki Bensaci, PhD, Merck: Employee Karri A. A. Bauer, PharmD, Merck: Employee Kenneth Klinker, PharmD, Merck: Employee Jason Cota, PharmD, Merck: Employee Pavel Prusakov, PharmD, Merck: Employee Rodrigo E. Mendes, PhD, GSK: Grant/Research Support|Shionogi & Co., Ltd.: Grant/Research Support|United States Food and Drug Administration: FDA Contract Number: 75F40123C00140 Kristen Feemster, MD, Merck: Employee

